# The effects of microplastics exposure on the growth and development of children

**DOI:** 10.3389/fpubh.2026.1755497

**Published:** 2026-03-25

**Authors:** Weifeng Xuan, Xiaomeng Yan, Shuping Zhao, Bo Qin

**Affiliations:** 1 Shaoxing Maternity and Child Health Care Hospital, Shaoxing, China; 2 Maternity and Child Health Care Affiliated Hospital, Shaoxing University, Shaoxing, China

**Keywords:** child growth and development, endocrine disruptors, gut microecology, health risk, hormonal regulation, microplastics

## Abstract

Microplastics (MPs), as emerging environmental pollutants, found from the deepest oceans to the highest mountains, and crucially, within the human body, have attracted increasing attention due to their widespread presence and potential health impacts. This review addresses the multifaceted influence of MP exposure on endocrine regulation, gut microecology, the potential transgenerational effects, and child growth and development. Firstly, the sources and environmental distribution characteristics of MPs were outlined, their persistence and bioaccumulation potential were highlighted. How MPs act as carriers of endocrine-disrupting chemicals, interfering with hormonal systems and potentially disrupting children’s physiological development was elucidated subsequently. Special emphasis is placed on the mechanisms by which MPs alter gut microbial communities, leading to dysbiosis that may compromise immune function and metabolic processes in children. By synthesizing recent advances in toxicology, microbiology, and pediatric research, present review amalgamates insights from contemporary studies, elaborates the comprehensive health risks posed by MP exposure during critical developmental periods, underscoring the urgent need for targeted preventive and regulatory measures to mitigate MP-related health hazards and promote child health. We aim to provide a scientific foundation for future research directions of MPs exposure and the development of effective intervention strategies.

## 1 Introduction

Children represent a unique population characterized by heightened vulnerabilities across physical, physiological, and psychological domains compared to adults. Child growth and development encompass a complex interplay of physiological, environmental, and psychosocial factors, and are commonly assessed using key physiological indicators such as height, weight, bone density, and neurodevelopmental milestones. The endocrine system plays a pivotal role in regulating these physiological processes through hormones such as growth hormone (GH), insulin-like growth factor-1 (IGF-1), thyroid hormones, and sex steroids, which orchestrate cellular proliferation, differentiation, and metabolic activities critical for somatic growth and neurodevelopment. GH and IGF-1 are central to linear growth and bone mineralization, while thyroid hormones influence brain maturation and metabolic rate. Disruptions in endocrine function, whether due to nutritional deficiencies, environmental toxins, or systemic inflammation, can adversely affect growth trajectories and developmental outcomes (1). The gut microbiota and intestinal ecosystem have emerged as critical modulators of child growth and development, influences nutrient absorption, immune system maturation, and metabolic programming (2). Dysbiosis or altered gut microbial composition has been linked to growth faltering, impaired cognitive development, and increased susceptibility to infections. Environmental exposures, including diet quality, sanitation, and chemical pollutants such as endocrine-disrupting microplastics (MPs), can perturb gut microbiota balance, thereby indirectly affecting child health outcomes. Interventions targeting gut health, including improved nutrition and hygiene practices, have demonstrated potential in enhancing growth and developmental indices (2).

MPs pollution has emerged as a pervasive and escalating global environmental challenge, characterized by the ubiquitous presence of plastic particles smaller than 5 millimeters in aquatic and terrestrial ecosystems. MPs originate from diverse sources, including the fragmentation of larger plastic debris, industrial effluents, agricultural practices, and consumer products, leading to their widespread dissemination in surface waters, sediments, soils, and biota, etc. (3). Ultraviolet radiation accelerates the aging of plastics, enhances the adsorption capacity of MP for pollutants and amplifies their toxicity (4). MPs have been detected in freshwater mussels, fish, and marine organisms worldwide, highlighting their capacity to bioaccumulate and biomagnify through trophic levels (5). The persistence and resistance of MPs to biodegradation exacerbate their environmental footprint, enabling them to serve as vectors for hazardous contaminants such as heavy metals, pesticides, and persistent organic pollutants, thus amplifying their ecological toxicity (6). Furthermore, MPs may facilitate the co-transport of environmental hormones (EH) and other toxicants into the gut, intensifying microbial perturbations and systemic toxicity (4). In agricultural ecosystems, MPs influence soil microbial communities and nutrient cycling, which indirectly affect plant growth and, through the food chain, human gut microbiota and health (7). MPs exposure adversely affects gut microbiota composition and function, leading to dysbiosis characterized by reduced microbial diversity, altered abundance of beneficial taxa, and compromised intestinal barrier integrity. Some gut microbiota in certain terrestrial organisms can break down MPs, while specific chemicals help alleviate the intestinal toxicity caused by MPs and nanoplastics (NPs) (4). Animal model studies have shown that MPs can induce gastrointestinal inflammation, disrupt microbial balance, and impair digestive enzyme activities, thereby threatening gastrointestinal health (8).

MPs were reported to infiltrate into human body through ingestion, inhalation, or dermal absorption (9), and detected in a variety of human tissue and biological samples including liver, lung, placenta, kidney, spleen, blood, sputum, and faeces, etc. (10). Children’s growth and development are highly sensitive to environmental influences, including exposure to MPs and associated endocrine disruptors. The interference of EH with hormonal signaling pathways can lead to profound effects on physical growth, neurodevelopment, and metabolic regulation in children. Maternal and early-life exposures to pollutants have been linked to altered developmental trajectories, with potential long-term consequences for cognitive function and mental health (11). The gut microbiota serves as a critical mediator in these processes, influencing nutrient metabolism, immune maturation, and neurodevelopmental outcomes during childhood and adolescence (12). Disruption of gut microbial homeostasis by MPs and EH may impair these developmental processes, contributing to increased risks of metabolic disorders, immune dysregulation, and neurobehavioral abnormalities. Socio-demographic factors and dietary habits during adolescence interact with gut microbiota composition, further modulating health outcomes (12). Maternal hormonal status and nutrition during pregnancy and lactation can influence infant growth patterns and neurodevelopment, highlighting the intergenerational impact of endocrine regulation (13).

MP pollution constitutes a multifactorial environmental threat with significant implications for endocrine regulation, gut microbial ecology, and children’s growth and development. MPs act as physical pollutants and vectors for EH and other toxicants, disrupting hormonal signaling and gut microbiota homeostasis. These disturbances can lead to adverse health outcomes in children, including impaired growth, metabolic dysregulation, and developmental disorders. Addressing these challenges requires an integrated approach encompassing environmental monitoring, mechanistic research, policy interventions, and public health strategies to mitigate MP exposure and its cascading effects on endocrine function, gut ecology, and pediatric health (14). This review aims to synthesize the intricate interplay among MP exposure, environmental hormone disruption, gut microbiota dysbiosis, and children’s growth, highlighting potential mechanisms and identifying critical knowledge gaps to guide future research and risk management efforts, aiming at protecting pediatric health in the context of escalating MP pollution (14).

### 1.1 Environmental sources of MPs and human intake pathways

#### 1.1.1 Classification and distribution of MPs

MPs are generally defined as plastic particles with sizes less than 5 millimeters, bottled drinking water, seafood, agricultural products, and ambient air have been identified as significant sources (15). The classification of MPs based on size is crucial for understanding their behavior, transport, and potential ecological impacts. MPs was further subdivided into large MPs (1–5 mm), small MPs (1 μm-1 mm), and NPs (<1 μm). There is a remarkably consistent relationship between size and abundance of MPs across different environmental matrices. While larger MPs pose ecological risks primarily through physical interactions and habitat disruption, NPs penetrate biological barriers more readily, eliciting cellular and molecular-level effects that may translate into population-level consequences. The surface area-to-volume ratio increases as particle size decreases, which directly influences the adsorption capacity and kinetics of MPs for co-contaminants. The size of MPs also plays a critical role, smaller particles, including NPs, often induce more severe gut microbiota perturbations and oxidative stress than larger MPs, as demonstrated in zebrafish and mice models (16, 17). The size-dependent effect extends to alterations in both bacterial and fungal communities, as well as metabolic pathways related to xenobiotic metabolism and immune modulation (16, 18). NPs, due to unique colloidal behavior and potential for cellular penetration, present distinct challenges and risks that are currently at the forefront of research in environmental toxicology (19). They can easily bind with natural colloids or organic matter, forming heterogeneous aggregates that alter their sedimentation rates and bioavailability, infiltrate deeper soil layers and potentially reach groundwater, posing a more insidious and long-term contamination risk. NPs remain suspended for extended periods, facilitated by ocean currents and wind, it allows them to migrate over vast distances, even reaching deep-sea environments and polar ice cores, where they can accumulate and pose risks to marine ecosystems, to exhibit more complex and heightened toxicological effects and particularly pronounced transgenerational effects (20).

**Figure 1:**
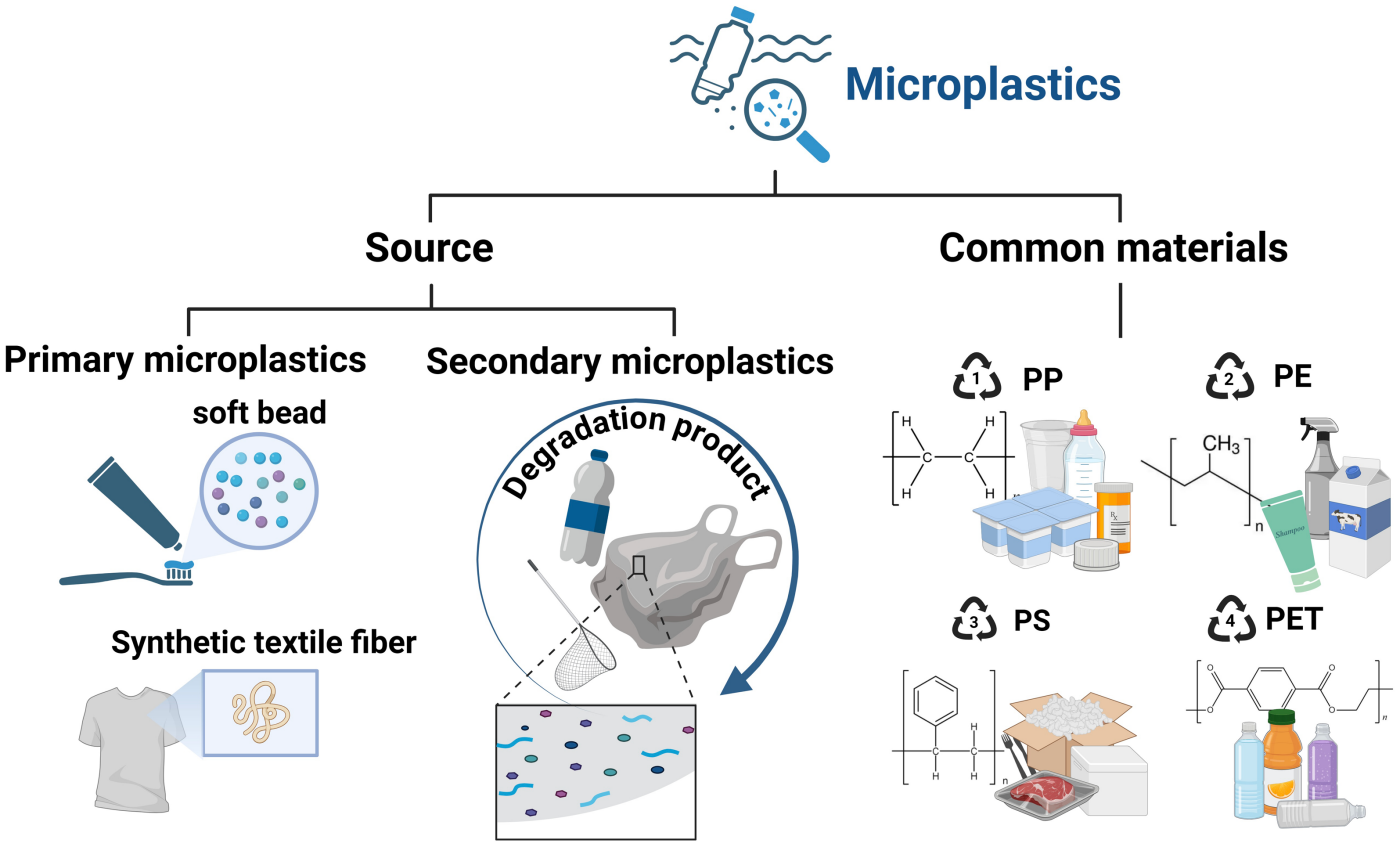
The classification and origin MPs. MPs were classified into primary and secondary MPs. Chemical composition of MPs includes PE, PP, PS, PET, and PA.

In China, intensive use of plastic mulch in agriculture, textile production, and daily consumer items has contributed to alarming concentrations of MPs in lakes, canals, and urban water bodies, posing significant health risks to local populations through contamination of drinking water and food chains (21). MPs exhibit diverse morphologies including fibers, fragments, films, pellets, spheres, rods, ellipses, and irregular shapes (22). The shape of MPs influences their environmental fate, transport, and biological interactions. As shown in Figure 1, MPs can further be classified into primary and secondary MPs. Primary MPs are intentionally manufactured small plastic particles used in products such as cosmetics, industrial abrasives, and seed coatings in agriculture. Secondary MPs arise from the fragmentation and weathering of larger plastic debris through mechanical, chemical, or biological processes in the environment. Chemical composition of MPs is equally varied, with common polymers including polyethylene (PE), polypropylene (PP), polystyrene (PS), PE terephthalate (PET), and polyamide (PA) (23). These polymers differ in density, hydrophobicity, and chemical reactivity, factors that influence their environmental behavior and interaction with pollutants such as heavy metals and organic contaminants (24). Due to irregular shape and reduced aggregation, secondary polyvinyl chloride (PVC) MPs are more toxic than primary ones (25). The distinction between primary and secondary MPs is critical for source identification and mitigation strategies. The chemical diversity and shape heterogeneity of MPs influence their interactions with environmental components, such as dissolved organic matter and heavy metals, affecting their transport and toxicity profiles (24).

MPs exhibit widespread and heterogeneous distribution across environmental compartments, driven by a combination of natural processes and anthropogenic influences. Their migration is mediated by hydrodynamic forces, atmospheric transport, and land use practices, with urban, agricultural, and industrial areas serving as key sources and hotspots. Understanding these distribution characteristics and migration mechanisms is essential for assessing ecological and human health risks and developing effective pollution control and remediation strategies (24). In aquatic environments, MPs have been detected globally in surface waters, sediments, and biota, spanning marine, freshwater, and estuarine systems. In terrestrial environments, MPs accumulate in soils and sediments, with concentrations varying widely depending on land use, proximity to pollution sources, and soil characteristics. Urban soils, agricultural lands, and landfill sites have been identified as hotspots of MP contamination, with polymers such as PE, PP, and PVC being predominant (26). The migration mechanisms of MPs involve physical, chemical, and biological processes. Physically, MPs undergo fragmentation, abrasion, and sedimentation; chemically, aging processes such as photo-oxidation and hydrolysis alter their surface properties and enhance pollutant adsorption; biologically, biofouling and microbial colonization modify particle density and reactivity, impacting transport and fate (27). MPs can adsorb various environmental pollutants, including heavy metals and residual antibiotics, which can be transported along with MPs, potentially increasing ecological and human health risks (28).

#### 1.1.2 Environmental persistence and bioaccumulation of MPs

The environmental persistence of MPs is a critical factor contributing to their widespread distribution and bioaccumulation in ecosystems. MPs are inherently resistant to degradation due to their polymeric structures. Their degradation in the environment is a slow process influenced by abiotic factors such as ultraviolet (UV) radiation, mechanical abrasion, and chemical weathering, as well as biotic factors. Recent studies have elucidated the formation of environmentally persistent free radicals (EPFRs) on MPs under UV irradiation, which alter their physicochemical properties and may affect their toxicity and environmental fate (29). UV exposure induces the generation of EPFRs on PS and PE MPs, with the type and concentration of radicals depending on the UV wavelength and polymer structure. These radicals can accelerate oxidative processes, leading to surface modifications but not necessarily complete mineralization, thus prolonging the MPs’ environmental persistence. EPFRs, reactive oxygen species (ROS) and oxidative potential derived from photoaging can alter the physicochemical properties of photoaged PS, they were reported to be able to shorten the longevity of *Caenorhabditis elegans* via inducing oxidative stress (30).

In various organisms ranging from invertebrates to fish and apex predators, MPs have been detected. The persistence of MPs facilitates their bioaccumulation and trophic transfer. Bioaccumulation studies in fish species such as medaka and tilapia demonstrate that MPs can accumulate primarily in the gastrointestinal tract with potential physiological impacts (31, 32). The bioaccumulation is influenced by factors such as particle size, shape, polymer type, and environmental aging, which affect the adsorption of co-contaminants like antibiotics, heavy metals, and persistent organic pollutants. Aged MPs exhibit enhanced adsorption capacities due to increased surface roughness and functional groups introduced during photoaging or microbial degradation, thereby acting as vectors that prolong the environmental persistence and bioavailability of these pollutants (33). Complex pollutants formed by degraded MPs and heavy metals can bioaccumulate through the food chain, ultimately posing significant threats to ecosystems and human health, often through the microbial loop. This type of combined pollution has emerged as a pressing global challenge requiring urgent attention.

MPs also interact with microbial communities by serving as substrates for biofilm formation, known as the plastisphere, which can harbor pathogens and antibiotic resistance genes (ARGs), further complicating their ecological impact and persistence (34). The biofilm and associated microbial activity can influence the degradation and fragmentation of MPs, generating secondary MPs and NPs that may have distinct environmental behaviors and toxicities. The transport and deposition of MPs are modulated by sediment composition, hydrodynamic forces, and biophysical flocculation processes, which determine their residence time and distribution in aquatic systems (35). The long-term environmental impact of MPs is also linked to their resistance to biodegradation. While certain microbial strains have been identified with the capacity to degrade MPs,the degradation rates are generally slow and incomplete, resulting in the continuous release of MPs and NPs into the environment. Surfactants were applicated to increase interfacial activation of enzyme and PET interactions. The persistence of MPs in soils and sediments is further compounded by their interactions with coexisting contaminants, which can alter soil microbial communities, nutrient cycling, and ecosystem functions (36).

### 1.2 Mechanisms of MPs affecting the growth and development of children

#### 1.2.1 The potential transgenerational effects of MPs on reproductive capacity

**Figure 2:**
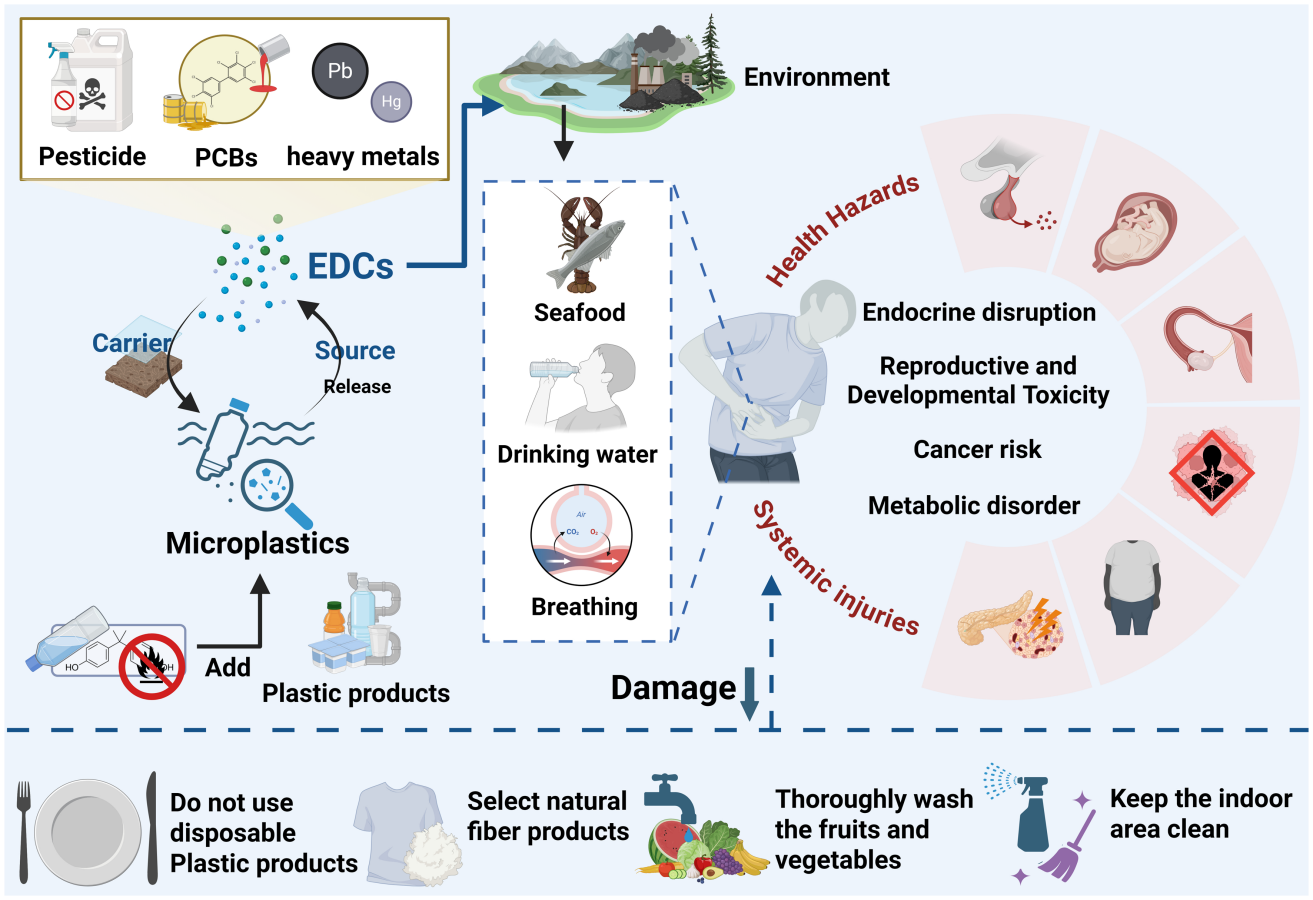
The mechanisms by which MPs affect children’s health and potential intervention measures.

MPs can translocate through the bloodstream and accumulate in reproductive organs, including testes, ovaries, and uterine tissues, affect the health of children by interfering with their parental’s reproductive systems (Figure 2). The male and female reproductive systems exhibit distinct anatomical, physiological, and hormonal characteristics that may influence their sensitivity to MPs. This differential accumulation pattern may be influenced by intrinsic biological factors such as hormonal milieu and structural differences between male and female reproductive organs. Experimental data from rodent models exposed to polystyrene MPs demonstrate that male rats experience more pronounced dyslipidemia and cardiovascular risks, which could indirectly affect reproductive health, whereas females show altered gut microbiota diversity linked to microplastic exposure, suggesting systemic sex-specific responses that may extend to reproductive tissues (37). MPs exposure can perturb the neuroendocrine axis, particularly the hypothalamic–pituitary-gonadal (HPG) axis, leading to altered sex hormone synthesis and reproductive dysfunctions in both males and females, including impaired spermatogenesis, placental abnormalities, and ovarian atrophy (38). Sex hormones like estrogen and testosterone regulate tissue permeability and immune responses, which could modulate microplastic retention and toxicity. The distinct architecture of the blood-testis barrier versus the ovarian follicular environment may dictate the localization and impact of MPs. Ecological studies in aquatic species such as crabs and squids reveal sex-based differences in microplastic load, with male crabs often exhibiting higher MP abundance potentially due to behavioral factors, while female crabs sometimes show greater contamination in certain habitats, underscoring the complexity of sex-related MP distribution across species (39).

Studies in mammalian models indicate that the male reproductive system exhibits heightened sensitivity to microplastic accumulation, especially manifesting as damage to the blood-testis barrier (BTB), which is crucial for maintaining an immune-privileged environment necessary for spermatogenesis, and its disruption by MPs can impair male fertility. MPs of nanoscale size accumulate extensively in testicular tissue and spermatogenic cells, whereas larger MPs show limited penetration and accumulation, which was correlated with impaired spermatogenic cell differentiation, reduced fertility, and disrupted epigenetic and metabolic pathways (40). NPs have been shown to penetrate cellular organelles such as mitochondria and lysosomes, inducing mitochondrial dysfunction, oxidative stress, and apoptosis in reproductive cells. The enhanced surface area and reactivity of MPs contribute to their ability to generate ROS, leading to oxidative damage, DNA damage, mitochondrial dysfunction, apoptosis and inflammatory responses within reproductive tissues (41). Male reproductive health risks characterized by reduced sperm count and motility were significantly associated with MP contamination, particularly polytetrafluoroethylene (PTFE) (21). The oocyte maturation and fertilization rate, embryo development, and fertility of mice were reduced significantly via oral administration of MPs for 30 days (40 mg/kg per day). Maternal exposure to MPs during pregnancy led to a decline in both birth and postnatal body weight of offspring mice (42). The presence of MPs in critical tissues like the placenta and their association with telomere shortening suggest possible impacts on biological aging and developmental programming, which could influence susceptibility to diseases in adulthood (43). Additionally, MP exposure has been linked to reproductive health impairments, including altered hormone levels and delayed reproductive maturity, which may have lifelong consequences (38). The disruptions of MPs have profound implications for offspring development and metabolic health, potentially extending across generations. The complexity of MP compositions and limitations in detection methodologies have constrained comprehensive understanding, underscoring the need for integrative studies that elucidate the mechanistic pathways of endocrine interference and reproductive toxicity (38).

#### 1.2.2 MPs interfering the gut microecology of children

The gut microbiota, an intricate and dynamic microbial community within the gastrointestinal tract, plays a pivotal role in host metabolism, immune regulation, nutrient metabolism, and overall health, particularly during critical windows of development. The homeostasis of gut microbial communities, consortium of bacteria, fungi, archaea, and viruses, is essential for nutrient absorption, barrier integrity, and modulation of systemic inflammatory responses, influencing the development of both innate and adaptive immunity, maintaining immune tolerance and protecting against pathogens (44). In childhood, the gut microbiota undergoes a critical developmental trajectory characterized by progressive diversification and stabilization during early life, influenced by mode of delivery, diet (breastfeeding versus formula feeding), environmental exposures, and antibiotic use (45, 46). The pediatric gut microbiota is particularly sensitive and plastic, with its composition and function susceptible to perturbations from environmental factors, including exposure to pollutants such as MPs. The gut microbiota’s diversity and function are central to immune regulation and nutrient metabolism, processes that are especially critical during childhood when the microbiota is still maturing and highly sensitive to environmental influences. The gut microbiota influences hormonal regulation and the gut-brain axis, factors crucial for neurodevelopment and systemic homeostasis. Perturbations in microbial communities during infancy have been linked to impaired nutrient absorption, increased intestinal permeability, and dysregulated immune responses, which may contribute to growth faltering and susceptibility to infections (46).

MPs, originating from industrial processes, consumer goods, and environmental degradation, have been detected in human consumables. Consequently, the gastrointestinal (GI) tract was identified as a primary route of human exposure. The fate of MPs after entering GI tract was up to the particles size. Smaller particles which cannot be excreted through the feces, will accumulate in gastrointestinal tract, particles with <150 microns in diameter can enter lymphatic and circulatory systems, accumulate in liver, kidneys, and brain eventually, bring a range of toxic effects (47). MPs could induce notable alterations in the gut microbial community structure, characterized predominantly by decreased microbial diversity, reduction of beneficial bacteria, and proliferation of harmful or pathogenic taxa, disrupt the production of critical microbial metabolites such as SCFAs, which are essential for maintaining intestinal barrier integrity and modulating inflammatory responses (18). Studies in mice exposed to PS-MPs have reported decreased alpha diversity indices, particularly in female mice, alongside significant shifts in beta diversity indicating altered microbial community composition (48). These changes often involve a decline in probiotic genera such as *Lactobacillus* and *Akkermansia*, coupled with an increase in potentially pathogenic bacteria including Escherichia-Shigella and Prevotellaceae (49). Similar patterns have been observed in terrestrial organisms, where MP exposure modulates gut bacterial communities by affecting key phyla like *Proteobacteria* and *Firmicutes*, and in honeybees, where PS-MPs reduce gut microbial diversity and alter core microbial populations (50, 51). Notably, the disruption of gut microbiota by MPs is often accompanied by physiological impairments such as intestinal barrier damage, inflammation, oxidative stress, and metabolic disturbances, suggesting a mechanistic link between microbial dysbiosis and host health detriments (52). Children are particularly vulnerable due to their higher intake of air, water, and food relative to body weight, coupled with behaviors such as hand-to-mouth activity and proximity to contaminated environments, which increase their exposure to MPs and associated toxins (53). Experimental animal studies have further elucidated mechanisms by which MP may contribute to metabolic dysregulation. For instance, prolonged ingestion of polystyrene MP has been shown to disrupt gut microbiota composition and induce systemic inflammation, which in turn can trigger ferroptosis, a form of iron-dependent cell death, in neuronal tissues, exacerbating neurological symptoms such as seizures. These findings highlight the interplay between inflammation and metabolic disturbances induced by MP exposure (53).

The extent and nature of microbiota disruption appear to be influenced by MP characteristics including polymer type, size, and concentration. Polystyrene MPs tend to induce more pronounced gut microbial dysbiosis and inflammatory responses compared to other polymers like PE or PP. (54, 55) Exposure to polystyrene MPs in aquatic organisms results in significant shifts in microbial taxa and reduced diversity, with concomitant intestinal damage exacerbated by environmental stressors like elevated temperature (55, 56). In contrast, polypropylene and high-density PE MPs may exert milder or differential effects on gut microbiota, as observed in earthworms where polypropylene exposure reduced bacterial diversity in soil but not significantly in the gut (50). Furthermore, aging and weathering of MPs can modify their surface properties and associated microbial communities, named plastisphere, potentially influencing their impact on host gut microbiota by serving as vectors for microbial transfer (44). Moreover, co-exposure to MPs and other contaminants such as heavy metals, antibiotics, or organic pollutants can synergistically exacerbate gut microbial dysbiosis and related health effects, highlighting the complexity of MPs impacts (48, 50).

In a word, as shown in Figure 2, MPs exposure disrupts gut microbiota structure by decreasing microbial diversity, depleting beneficial probiotic populations, and promoting the growth of harmful bacteria, which collectively contribute to host intestinal dysfunction and systemic health risks. Disruptions of MPs exposure to delicate microbial ecosystem of children may have more profound effects on pediatric health outcomes. The severity and specific patterns of microbiota alterations depend on MP type, size, concentration, and co-exposure to other pollutants. Understanding these differential effects is crucial for elucidating the mechanisms underlying MP-induced gut dysbiosis and for developing targeted interventions to mitigate their adverse health consequences. Future research should focus on characterizing the specific microbial taxa and metabolic pathways affected by such exposures in children, and on developing interventions to preserve or restore gut microbial homeostasis during this vulnerable period.

#### 1.2.3 MP exposure perturbs the gut metabolites and immune function of children

MPs exposure has been increasingly recognized as a significant environmental factor altering gut metabolic profiles, particularly affecting key metabolites such as short-chain fatty acids (SCFAs) that are crucial for maintaining intestinal and systemic health. SCFAs, including acetate, propionate, and butyrate, are primarily produced by microbial fermentation of dietary fibers and play vital roles in modulating immune function, energy metabolism, and gut barrier integrity, especially in adolescents (12). MPs exposure disrupts the production and balance of these metabolites by perturbing gut microbial communities (57). In mouse models, polystyrene MP exposure led to a significant decrease in butyrate levels, especially under high-fat diet conditions, which correlated with impaired intestinal barrier function and altered expression of tight junction proteins and peroxisome proliferator-activated receptor *γ* (PPARγ) signaling pathways (58). This reduction in butyrate, a key anti-inflammatory metabolite, may contribute to increased gut permeability and systemic inflammation, thereby exacerbating metabolic disorders. Metabolomic analyses in various animal models consistently reveal that MPs induce broad alterations in gut metabolites involved in lipid metabolism, amino acid metabolism, and energy homeostasis. Exposure to environmental concentrations of MPs in silkworms disrupted metabolites related to energy and lipid metabolism, xenobiotic detoxification, and immune responses, suggesting a direct impact on physiological and metabolic states (59). Zebrafish exposed to PE and polyester MPs showed significant changes in metabolites linked to lipid signaling molecules and amino acid metabolism, accompanied by gut microbiota dysbiosis (60).

*In vitro* studies simulating human colon conditions have demonstrated that exposure to these MPs induces an overgrowth of opportunistic bacteria, including *Enterobacteriaceae*, *Desulfovibrio* spp., and *Clostridium* groups, while concurrently reducing beneficial bacterial taxa except for *Lactobacillales*. This dysbiotic shift is accompanied by changes in bacterial metabolic activity, suggesting that MP ingestion may disrupt the delicate balance of the gut microbiome, which is crucial for immune homeostasis and metabolic regulation (61). Atmospheric MPs have been linked to increased pathogenicity of airborne bacterial communities, with certain bacterial genera like *Sphingomonas* possibly mediating immune-related disease risks (62). It suggests that MP exposure may enhance the burden of immune-mediated diseases through both direct and indirect pathways involving microbial interactions.

The interaction between MPs and co-exposed environmental pollutants further complicates the metabolic outcomes. MPs can enhance the bioavailability of heavy metals by altering gut microbial metabolites, including amino acid derivatives and organic acids, which increase metal solubility in the intestinal lumen (63). The synergistic effect may exacerbate toxic metal accumulation and disrupt host metabolism. Combined exposure to MPs and antibiotics like tetracycline altered gut microbial networks and metabolic pathways, affecting protein digestion and absorption, which could impair nutrient utilization and immune function (64). Sex-specific differences in metabolite alterations have also been observed. Co-exposure to polystyrene MPs and Lead(II) caused distinct changes in fecal metabolites between male and female mice, with females exhibiting more pronounced reductions in microbial diversity and metabolite profiles linked to immune and metabolic pathways (48). The release of additives from MPs in the gut environment has been shown to inhibit microbial metabolic activity and reduce SCFA production, particularly affecting the luminal microbiota more than the mucosal microbiota. It may impair gut homeostasis and contribute to intestinal inflammation (57). The gut microbiota-mediated degradation of certain biodegradable MPs can also exacerbate metabolic dysregulation by altering lipid metabolism and intestinal barrier function (65). The immune and metabolic systems of children are still immature and developing, alterations in gut metabolites induced by MPs exposure could have profound implications. Disturbances in the level of SCFAs which can regulate immune maturation and metabolic programming, may predispose children to immune dysregulation, increased susceptibility to infections, and metabolic disorders such as obesity and insulin resistance. The modulation of gut metabolites by MPs may also influence the gut-liver axis, contributing to hepatic inflammation and metabolic syndrome (66).

#### 1.2.4 MP-induced intestinal barrier dysfunction

MPs can accumulate in the human body primarily through ingestion, posing significant threats to intestinal barrier integrity. The intestinal epithelial barrier comprises a mucus layer, epithelial cells connected by tight junctions (TJs), and immune components, all of which maintain selective permeability and protect against harmful substances. MPs disrupt this barrier through both mechanical and chemical mechanisms. Mechanically, MPs can physically interact with and damage the epithelial surface, leading to histopathological alterations such as epithelial cell injury and reduced mucus secretion. Exposure to PS-MPs in animal models has been shown to cause mucosal damage, decreased mucin secretion, and disruption of TJ proteins including occludin, claudin-1, and zonula occludens-1, which are critical for maintaining epithelial tightness (67), intestinal permeability increased, facilitating the translocation of MPs and other luminal antigens into systemic circulation. Chemically, MPs can induce oxidative stress and inflammatory responses within the gut mucosa. The generation of ROS triggered by MPs activates signaling pathways such as NF-κB and the NLRP3 inflammasome, leading to the upregulation of pro-inflammatory cytokines like interleukin-1β (IL-1β) and tumor necrosis factor-alpha (TNF-*α*) (67, 68). This inflammatory milieu further damages the epithelial barrier by promoting apoptosis of epithelial cells and disrupting TJ protein expression. Additionally, MPs can adsorb and carry environmental pollutants such as heavy metals and persistent organic pollutants, exacerbating their toxic effects on the intestinal barrier (69). The combined exposure to MPs and contaminants like cadmium or perfluorooctanoic acid (PFOA) has been shown to synergistically impair barrier function, enhancing oxidative stress and inflammation (69).

The compromised intestinal barrier caused by MPs not only facilitates their systemic absorption but also initiates local and systemic immune responses. Increased permeability allows luminal bacteria and endotoxins to penetrate the mucosa, triggering immune cell infiltration and chronic inflammation, and implicated in the exacerbation of inflammatory bowel diseases (IBD) and contributed to metabolic and hepatic disorders via the gut-liver axis (68). MPs-induced barrier dysfunction has been linked to alterations in gut microbiota composition, characterized by dysbiosis with an increase in pathogenic bacteria and a decrease in beneficial microbes, which further perpetuates inflammation and barrier impairment (59). In C57BL/6 mice, oral administration of PS-MPs increased intestinal permeability, decreased mucus secretion, and downregulated TJ proteins, effects that were more pronounced when combined with dietary factors such as high-fat diet or alcohol consumption (58). *In vitro*, exposure of human intestinal epithelial cells to true-to-life MPs resulted in compromised barrier integrity and elevated inflammatory cytokine secretion. Protective agents like melatonin have shown potential in reversing MP-induced barrier damage by reducing oxidative stress and restoring TJ protein expression (59). Understanding these mechanisms underscores the critical need for strategies to mitigate MP exposure and protect intestinal barrier integrity to safeguard human health.

#### 1.2.5 MPs threaten children by serving as carriers of EH

EH, commonly referred to as endocrine-disrupting chemicals (EDCs), encompass a diverse group of synthetic and natural compounds. Among the most prevalent EDCs are bisphenol A (BPA), phthalates (notably phthalic acid esters, PAEs), and polychlorinated biphenyls (PCBs). BPA, phthalates, and PCBs exert endocrine disruption through their chemical structures that enable them to mimic or block endogenous hormone signals, so as to achieve the purpose of receptor binding interference, modulation of hormone biosynthesis enzymes, and alteration of hormone metabolism (70). BPA, a phenolic compound, is widely used in the production of polycarbonate plastics and epoxy resins, mimics the natural hormone estradiol via binding estrogen receptors (ERs), leads to aberrant activation or suppression of estrogen-responsive genes, thereby disrupts physiological processes regulated by estrogen signaling. BPA and its analogs have been shown to induce oxidative stress and DNA fragmentation, highlighting their cytotoxic potential beyond receptor-mediated actions (71). Phthalates, particularly di(2-ethylhexyl) phthalate (DEHP), are plasticizers incorporated into PVC and other polymers to enhance flexibility. Co-exposure of PS-MPs and DEHP evoked oxidative stress which led to the activation of NF-κB/NLRP3 pathway and kidney pyroptosis (72). Lipophilic properties facilitate the bioaccumulation of phthalates, which disrupt endocrine primarily by interference with steroidogenesis, by binding to and antagonizing estrogen and androgen receptors, as well as by modulating enzymes critical for hormone biosynthesis. DEHP acts as an antiestrogen by binding to estrogen receptors, suppressing gene expression of steroidogenic enzymes like CYP3 and 17β-HSD, leading to reduced estradiol and progesterone levels and consequent reproductive impairment (73). Phthalates can be adsorbed onto MPs, facilitating their environmental transport and increasing exposure risk (74). PCBs with high chemical stability and lipophilicity are persistent organic pollutants composed of biphenyl molecules chlorinated at various positions. PCBs disrupt endocrine function primarily through structural mimicry of thyroid hormones and interaction with thyroid hormone receptors (THRs), leading to altered thyroid hormone synthesis, metabolism, and signaling, disturb developmental and metabolic processes regulated by thyroid hormones (75). PCBs also exhibit estrogenic and antiandrogenic activities, contributing to a broad spectrum of endocrine disruptions. Their persistence and bioaccumulation in aquatic and terrestrial food webs pose significant risks to wildlife and human health (76).

MPs not only carry intrinsic chemical additives but also adsorb and release EH and other pollutants, thereby acting as vectors that facilitate the bioavailability of endocrine disruptors in organisms (38). The desorption of EH from MPs is contingent upon temperature, pH, hydrophobicity, and the physicochemical characteristics of both MPs and adsorbed compounds. Temperature and diffusion pathways further modulate desorption kinetics, with the intestinal environment of warm-blooded organisms identified as a site of highest bioavailability for PAEs released from MPs. It suggests that MPs can act as reservoirs, delivering adsorbed EDCs into biological systems where they may exert toxicological effects (74). Wastewater treatment plants have been identified as significant sources releasing both MPs and associated EDCs into aquatic systems. The correlation between MPs and EDC concentrations implies a shared origin and highlights the potential for MPs to release EDCs during treatment processes, contributing to environmental contamination and posing risks to aquatic organisms (77). Emerging contaminants adsorbed on MPs include a broad spectrum of compounds such as UV filters, steroid hormones, and pharmaceuticals, with concentrations in the nanogram per gram range detected even in remote marine environments. PS-MPs exhibit increased charge distribution upon aggregation in aqueous environments, generating permanent dipoles that enhance their solubility and facilitate the adsorption of EDCs such as ethynylestradiol, estradiol, and BPA with adsorption energies exceeding 15 kcal/mol. Environmental factors such as aging and photo-oxidation of MPs significantly influence their surface properties and consequently their adsorption behavior. PE microfibers subjected to UV-C irradiation exhibit increased surface roughness and the formation of oxygen-containing functional groups, which reduce hydrophobicity from approximately 80° to 65° as evidenced by decreased contact angles. This alteration leads to diminished adsorption rates of hydrophobic hormones like 17α-ethynylestradiol (EE2), indicating that aging processes modulate the ecological risk profile of MPs by altering their capacity to adsorb and transport EH (78). The presence of biofilms and weathering can modify MPs’ surface chemistry, potentially influencing their interaction with endocrine disruptors in aquatic systems.

The presence of chemical additives intrinsic to MPs, including plasticizers like phthalates and bisphenols, further complicates their role as carriers of endocrine disruptors. These additives can leach from MPs under various environmental conditions, contributing directly to endocrine disruption. Experimental evidence from marine mussels exposed to high-density PE MPs and DEHP reveals molecular initiating events such as binding of DEHP to estrogen receptors, suppression of steroidogenic enzymes, and consequent impairment of reproductive function. This demonstrates that MPs not only adsorb external EDCs but also release intrinsic additives that act as EH (73). The mechanism by which MPs act as carriers of EH involves initial adsorption driven by physical interactions modulated by MPs’ surface properties and environmental aging processes, followed by conditional desorption influenced by environmental and physiological factors. This dual role as vectors and sources of endocrine disruptors amplifies the ecological and health risks posed by MPs, bringing challenges in mitigating their impacts on children and ecological health, necessitating further research into their behavior in diverse environmental matrices and biological systems.

#### 1.2.6 Synergistic exposure to MPs and EH on children

Co-exposure to MPs and EH or EDCs can exert synergistic adverse effects on the endocrine system. A study exposing mice to environmentally sourced PVC MPs containing multiple additives, including seven known endocrine disruptors, demonstrated increased molecular markers of inflammation and oxidative stress in intestinal tissues, exacerbating disease states such as chronic colitis (79). This suggests that MPs can act as vectors delivering EDCs into biological systems, potentially amplifying toxicological effects. Juvenile *Cyprinus carpio* exposed to PE MPs combined with 4-nonylphenol (4-NP), a potent alkylphenol endocrine disruptor, showed significant alterations in blood biochemical and hematological parameters, indicating systemic toxicity and highlighting the synergistic impact of MPs and EH (80). The potential risks of such synergistic effects are particularly concerning for children, whose endocrine systems are highly sensitive during critical developmental windows. Early-life exposure to MPs laden with endocrine disruptors has been linked to altered hormone receptor expression, immune dysfunction, and developmental abnormalities. Bisphenols and phthalates released from plastics have been implicated in modulating neuroendocrine pathways and immune responses during perinatal stages, which may predispose children to reproductive and neurological disorders later in life (81). MPs combined with steroidal estrogens or other EDCs can upregulate inflammatory and hormone receptor genes, exacerbating endocrine disruption in fish models (82). Given that MPs can cross biological barriers and have been detected in blood and placenta, combined exposure to MPs and EH may impair endocrine function in children, potentially affecting growth, sexual maturation, and metabolic regulation (83). Accompanied with the production increase of plastics, the prevalence of obesity has elevated in recent decades. MPs affected adipocyte differentiation via oxidative stress following accumulation in liver and kidney and alterations in energy balance and lipid metabolism (84). The mRNA levels of key genes involved in lipogenesis and triglyceride synthesis were decreased after exposure to PS MPs in mice (85). Therefore, the synergistic interaction between MPs and EH represents a multifaceted threat to the pediatric endocrine system, necessitating comprehensive mechanistic studies and epidemiological investigations to elucidate exposure pathways, dose–response relationships, and long-term health outcomes. Preventive strategies should prioritize reducing environmental release of MPs and EDCs, improving wastewater treatment technologies, and monitoring vulnerable populations, especially children, to mitigate these emerging risks.

#### 1.2.7 Endocrine disruption effects of MPs on child development

MPs can carry and release EDCs which are known to mimic or block natural hormones, thereby disturbing the endocrine system, leading to developmental abnormalities including precocious puberty and growth retardation (86). The vulnerability of children to these disruptions is heightened by their ongoing physiological development and the sensitivity of their endocrine systems to exogenous chemical interference (71). Epidemiological evidence supports the association between exposure to plastic-associated EDCs and adverse developmental outcomes in children (71). Exposure dose–response relationships indicate that higher environmental and internal doses of EDCs correlate with increased risks of hormonal imbalances manifesting as early onset of puberty or delayed growth milestones. The Minderoo-Monaco Commission report highlights that infants and children are particularly susceptible to plastic-associated chemical exposures, which are linked to prematurity, low birth weight, neurodevelopmental impairments, and reproductive organ malformations. Moreover, the complexity of plastic chemical mixtures and their persistence in the environment exacerbate exposure risks, as children may encounter multiple EDCs simultaneously, leading to additive or synergistic effects on endocrine function (77). The epidemiological data consistently demonstrate that even low-level chronic exposure to MPs and their endocrine-disrupting cargos can have significant implications for hormonal regulation and developmental trajectories in children (87). This necessitates urgent attention to environmental sources of MPs and EDCs, improved monitoring of exposure levels, and comprehensive risk assessments to elucidate the full spectrum of health outcomes.

### 1.3 Prevention and intervention strategies of children’s health

#### 1.3.1 Reducing MP exposure: environmental and policy measures

Internationally and domestically, a variety of policies and regulations have been enacted to curb plastic use and MP emissions, reflecting growing awareness of their environmental and health impacts. Israel has implemented several laws aimed at reducing plastic production and waste, including taxes on single-use plastics (SUPs) and standards for food-, beverage-, and child-safe plastics. China has recognized the need for standardized protocols and risk management strategies to control MP pollution, encouraging systematic research and policy development to address the diverse sources and ecological consequences of MPs (88). In European, econometric models integrating circular economy tools have been proposed to enhance the efficiency of water pollution control policies related to MPs, emphasizing the importance of data-driven decision-making (89). Southeast Asian countries face challenges due to rapid urbanization and plastic waste mismanagement. Bangladesh has seen a significant increase in plastic consumption and waste, prompting calls for integrated approaches combining research, policy enforcement, and technological innovation to mitigate MP pollution (90).

Environmental remediation technologies complement policy measures by targeting MP removal and degradation. Advanced WWTPs have been evaluated for their MP removal efficiencies, with secondary and tertiary treatments showing varying degrees of success. Life cycle assessments indicate that MP emissions from WWTPs significantly contribute to environmental impacts, underscoring the need for improved treatment technologies and operational strategies (77). Biodegradation approaches employing microorganisms such as Penicillium brevicompactum have demonstrated potential in fragmenting and degrading MPs from electronic waste, offering sustainable remediation pathways (91). Additionally, biochar derived from biomass wastes has emerged as a promising adsorbent for MPs and other pollutants in water, with ongoing research focusing on optimizing preparation methods and pollutant removal efficacy (92). On the prevention front, innovations in material science, such as modifying PA fibers with polydimethylsiloxane to reduce microfiber shedding during laundry, represent effective strategies to limit MP release at the source (93).

Public education is critical to complement regulatory and technological efforts. Studies emphasize the importance of educating the public on the environmental and health risks posed by MPs, fostering behavioral changes that reduce plastic consumption and improve waste management practices. International cooperation and open collaboration among scientists, regulators, and policymakers are essential to overcome the complexities of MP pollution (94). The COVID-19 pandemic has further complicated the landscape, increasing single-use plastic consumption and challenging waste management systems worldwide, thereby necessitating adaptive policies and enhanced public engagement to mitigate secondary MP generation (95). Effective reduction of MP exposure requires a multifaceted approach integrating stringent and enforceable policies, advanced environmental remediation technologies, and comprehensive public education. National and international regulatory frameworks must evolve to address the full spectrum of MP sources, including emerging contaminants like biodegradable MPs, while fostering innovation in material design and waste treatment. Coordinated efforts across sectors and regions will be vital to safeguard environmental and human health from the pervasive threats posed by MP pollution.

#### 1.3.2 Clinical and nutritional interventions

The protection of children’s health in the context of MP exposure and EDCs necessitates a multifaceted clinical and nutritional intervention approach, with a particular focus on restoring and maintaining gut microbial balance and early detection of harmful exposures. Nutritional strategies aimed at improving gut microbiota composition have garnered attention due to the gut microbiome’s critical role in modulating immune function, metabolic processes, and endocrine regulation, all of which can be disrupted by MP-associated EDCs. The administration of prebiotics and probiotics constitutes a promising nutritional intervention to enhance gut microbial diversity and resilience. Prebiotics, non-digestible food components, selectively stimulate the growth and activity of beneficial bacteria, while probiotics introduce live beneficial microorganisms to the gut ecosystem. Clinical trials have demonstrated that such interventions can mitigate inflammation and improve metabolic and neurodevelopmental outcomes in children, potentially counteracting the dysbiotic effects induced by MP exposure (96).

Clinical vigilance through systematic monitoring of EDC exposure is critical. Early screening programs aimed at detecting biomarkers of endocrine disruption can facilitate timely interventions to prevent or reduce adverse health outcomes. Given the ubiquitous presence of MPs and their potential to carry EDCs, routine surveillance in pediatric populations, especially those in high-risk environments, is imperative. This includes biomonitoring of MP-related chemicals in biological samples, as well as assessment of neurodevelopmental and endocrine function (87). The integration of community health workers into pediatric care teams has been shown to enhance preventive care delivery and parental guidance, which can be leveraged to incorporate exposure monitoring and education on minimizing contact with endocrine disruptors. Digital and home-based interventions, including internet-based mental health support and behavioral coaching, can complement clinical efforts by addressing psychosocial stressors that may exacerbate vulnerability to environmental toxins (87, 96). Clinical and nutritional interventions for protecting children’s health amid MPs and endocrine disruptor exposure should prioritize gut microbiota restoration through prebiotic and probiotic supplementation, implement systematic exposure monitoring and early screening protocols, and engage multidisciplinary approaches that include community health workers and digital health tools.

#### 1.3.3 Advances in techniques for studying MPs and endocrine disruptors

The investigation of MPs and their interactions with EDCs has evolved significantly, driven by the need for sensitive detection and comprehensive exposure assessment methods. High-sensitivity analytical techniques such as atmospheric pressure gas chromatography-tandem mass spectrometry (APGC-MS/MS) have been developed to quantify trace levels of PAEs, a common class of EDCs, in complex matrices and to elucidate their interactions with polyvinyl chloride MPs (PVC MPs) (74). It offers rapid, reliable, and sensitive detection with limits of detection as low as 0.0025 μg/L, enabling detailed mechanistic studies of adsorption and desorption processes influenced by hydrophobic interactions and van der Waals forces. Chromatographic techniques coupled with mass spectrometry have been optimized to detect a broad spectrum of plastic additives, including phthalates, terephthalates, and other plasticizers, in environmental samples such as settled dust, serving as markers for MP contamination (97). These advances facilitate the identification of bioaccumulative and potentially reprotoxic endocrine disruptors associated with MPs in various environments.

Emerging multi-omics approaches, including transcriptomics, metabolomics, and proteomics, are increasingly applied to unravel the complex mechanisms underlying MPs and EDCs toxicity. Transcriptomic analyses have revealed gene expression changes related to oxidative stress, inflammation, and endocrine pathways in organisms exposed to MPs and plasticizers (73, 76). Metabolomics profiling has identified disruptions in key metabolic pathways, such as the kynurenine pathway and lipid metabolism, following exposure to PA MPs and associated chemicals, linking molecular alterations to immunotoxic and endocrine outcomes (98). These omics techniques provide comprehensive datasets that facilitate the identification of biomarkers and mechanistic pathways involved in MP-induced endocrine disruption. The integration of multi-omics with advanced imaging and high-throughput screening technologies holds great promise for elucidating the interactive effects of MPs and EDCs at molecular, cellular, and organismal levels. Computational chemistry and molecular modeling have also contributed mechanistic insights into the adsorption behaviors of MPs with various endocrine disruptors, highlighting the roles of electrostatic and dispersion forces in pollutant sorption and cotransport in aquatic environments (71). Such mechanistic understanding is critical for predicting environmental fate and biological impacts.

*In vivo* exposure assessments have also progressed, with studies employing environmentally sourced MPs to better mimic real-world conditions. Subchronic ingestion of polyvinyl chloride MP fragments containing multiple additives, including known endocrine disruptors, has been shown to induce intestinal inflammation and fibrosis in murine models, highlighting the importance of using environmentally relevant particles for exposure evaluation (79). Laser direct infrared (LD-IR) chemical imaging combined with liquid chromatography–tandem mass spectrometry (LC–MS/MS) has been utilized to quantify placental MP burdens and correlate them with alterations in fetal hormone levels, providing a novel approach to assess in utero exposure and endocrine disruption (99). These integrative analytical strategies enable the simultaneous detection of MP polymers and associated hormonal changes, offering insights into potential developmental impacts.

## 2 Conclusion

It is clear that MPs do not merely pose a physical threat to ecosystems but also serve as vectors for EH, which can profoundly interfere with the delicate endocrine systems of developing children. This dual role amplifies the complexity of their health implications, necessitating a balanced interpretation of diverse research findings. The disruption of gut microbiota and impairment of intestinal barrier integrity by MP exposure represent critical mechanistic pathways linking environmental contamination to systemic health effects. These alterations compromise immune function and metabolic regulation, thereby increasing susceptibility to a spectrum of pediatric health issues. Moreover, the synergistic interaction between MPs and endocrine-disrupting chemicals further exacerbates risks of growth and developmental abnormalities, including hormonal imbalances, delayed maturation, and heightened predisposition to metabolic disorders. Such convergence of factors highlights the importance of integrating microbiological, endocrinological, and toxicological perspectives to fully elucidate the pathophysiological consequences. In conclusion, the burgeoning evidence on MPs as pervasive environmental pollutants underscores their multifaceted impact on children’s health, particularly through endocrine disruption and gut microbiome perturbations.

Environmental remediation efforts aimed at reducing MP pollution must be coupled with stringent policy frameworks that regulate plastic production, usage, and waste management. Concurrently, clinical interventions focusing on early detection and mitigation of endocrine and metabolic disturbances in exposed children are essential. Interdisciplinary collaboration among environmental scientists, clinicians, policymakers, and public health experts will be pivotal in crafting effective prevention and intervention paradigms. Despite these advances, current research on MP exposure in children remains limited by gaps in mechanistic understanding and a paucity of longitudinal epidemiological data. The heterogeneity of study designs, exposure assessment methods, and outcome measures complicates the synthesis of findings and calls for standardized protocols. Future investigations must prioritize comprehensive mechanistic studies that delineate causal pathways, alongside robust long-term cohort studies to capture the chronic and cumulative effects of MP exposure throughout critical windows of child development. In essence, safeguarding children’s health in the era of pervasive MP pollution requires a nuanced, evidence-based approach that reconciles diverse research perspectives. By advancing mechanistic insights, enhancing epidemiological rigor, and implementing integrated environmental and clinical strategies, the medical and scientific communities can better mitigate the insidious impact of MPs. This concerted effort will ultimately contribute to ensuring healthier developmental trajectories and improved long-term outcomes for future generations.
